# Performance of pyrosequencing versus MALDI-TOF MS in bacteria identification in chronic lung disease

**DOI:** 10.14440/jbm.2016.125

**Published:** 2016-08-13

**Authors:** Lucie Navrátilová, Petra Procházková, Jan Bardoň, Radko Novotný, Martin Zápalka, Petr Jakubec, Jaromír Zatloukal, Vítězslav Kolek, František Kopřiva, Pavla Flodrová, Vladislav Raclavský

**Affiliations:** ^1^Department of Microbiology, Faculty of Medicine and Dentistry, Palacký University Olomouc, Hnevotinska 3, 779 00 Olomouc, Czech Republic; ^2^Laboratory of Growth Regulators, Palacký University Olomouc & Institute of Experimental Botany AS CR, Slechtitelu 27, 783 71 Olomouc, Czech Republic; ^3^State Veterinary Institute Olomouc, Jakoubka ze Stribra 1, 779 00 Olomouc, Czech Republic; ^4^Department of Pediatrics, University Hospital Olomouc, I. P. Pavlova 6, Olomouc, Czech Republic; ^5^Department of Respiratory Medicine, University Hospital Olomouc, I. P. Pavlova 6, Olomouc, Czech Republic; ^6^Institute of Molecular and Translational Medicine, Hnevotinska 5, Olomouc, Czech Republic; ^7^Department of Clinical and Molecular Pathology, Faculty of Medicine and Dentistry, Palacký University Olomouc, Hnevotinska 3, 779 00 Olomouc, Czech Republic

**Keywords:** MALDI-TOF MS, pyrosequencing, 16S rDNA, Cystic fibrosis, Chronic obstructive pulmonary disease

## Abstract

Rapid identification of the etiological agent in bacterial infection is necessary for correct diagnosis and appropriate therapy. In general, identification of pure cultures of bacteria using conventional phenotyping techniques requires 4-24 hours. Recently available new molecular technologies offer the potential of same day species identification once pure culture is available. Our aim was to evaluate the performance of rDNA V1 hypervariable region pyrosequencing, and the whole cell MALDI-TOF MS protein profiling in routine species identification. During the period from June 2012 to June 2014, 1.140 pure culture isolates were recovered from 402 samples from 126 patients suffering cystic fibrosis, chronic obstructive pulmonary disease or bronchiectasis. All the isolates were subjected to species identification by both techniques. Unfortunately, pyrosequencing was able to reach the species level in 43.2% of isolates only, whereas MALDI-TOF was clearly superior with 96.8% respectively. The overall sensitivity values also clearly underlined the superiority of MALDI-TOF MS with 96.8% compared to 85.1% achieved by pyrosequencing. Generally, MALDI-TOF MS turned out to be the best suitable technique in routine bacterial identification, whereas pyrosequencing could be recommended as the method of choice particularly in situations where MALDI-TOF MS fails to identify rare species.

## INTRODUCTION

For clinical practice it is crucial to define the infectious agent as quickly and precisely as possible. Especially severe and rapid progressive diseases such as sepsis with high mortality rates need to be diagnosed properly and without latency. Molecular techniques based on direct detection of a particular gene using typically PCR combined with probe hybridization or sequencing may solve this extremely important medical problem [1, 2, 3]. However, conventional routine bacterial identification still relies on cultivation followed by identification of pure culture in most of the other samples. The rationale of this is not only the economy and high throughput of cultivation, but also frequent need of phenotypic antibiotic susceptibility testing on pure culture. Therefore, species identification techniques that are universally applicable on pure cultures of a wide spectrum of bacteria will most probably continue to prevail in routine diagnostic laboratories in the foreseeable future. In the recent years, whole cell MALDI-TOF MS profiling has been implemented in most diagnostic laboratories [2]. Next-generation sequencing that offers reduced costs compared to traditional Sanger sequencing, represents another potentially broadly applicable alternative.

The matrix assisted laser desorption/ionization-time of flight mass spectrometry (MALDI-TOF MS) identification is based on individual characteristic patterns of highly abundant proteins that are found in all microorganisms. The detected pattern is being matched with an extensive open database, which allows for reliable and accurate identification of the particular microorganism [4, 5].

The pyrosequencing is a powerful sequencing and quantification platform for microbial identification, which could namely be positively influenced by proper and adequate selection of the individual examined DNA region [6, 7]. For economic reasons, however, not more than 1 or 2 regions can be sequenced to keep costs competitive compared to MALDI-TOF MS. The aim of this study was to evaluate the performance of a single pyrosequencing reaction targeting the V1 hypervariable region of the 16S rRNA gene in quasi-routine settings, compared to routine MALDI-TOF MS protein profiling.

## MATERIALS AND METHODS

During the period from June 2012 to June 2014, 402 respiratory pathways samples obtained from 126 patients suffering chronic lung disease (cystic fibrosis, bronchiectasis and chronic obstructive pulmonary disease) were collected. Primary cultivation was done on Columbia blood agar (Oxoid) and *Haemophilus* selective agar (Oxoid) at 37°C in 5% CO_2_ atmosphere; on McKay agar (Oxoid) at 37°C under anaerobic condition (80% N_2_, 10% H_2_, 10% CO_2_); and on *Burkholderia cepacia* agar (Oxoid) at 30°C in ambient air, yielding 1.140 clinical isolates of bacteria altogether. The obtained isolates were accompanied by 8 reference strains (*Burkholderia multivorans* LMG 13010, *B. cenocepacia* LMG 16656, *Enterococcus faecalis* CCM 4224, *Escherichia coli* CCM 3954, *Pseudomonas aeruginosa* CCM 3955, *Staphylococcus aureus* CCM 3953 and CCM 4223, *Streptococcus mitis* CCM 7411 and *S sanguinis* CCM 4047) for functional verification of the methods.

Whole cell MALDI-TOF MS protein profiling was performed on a Microflex LT/SH system (Bruker, Germany) with HCCA matrix (α-Cyano-4-hydroxycinnamic acid, Sigma-Aldrich). A MALDI Biotyper software was used for species identification in accordance with manufacturer’s instruction. Template DNA was extracted using PathogenFree DNA PCR Kit (GeneProof, Czech Republic) according to the manufacturer’s instructions. The V1 hypervariable region of the 16S rDNA was amplified using primers bio-pBR5`.SE and pBR-V1.AS [7]. Briefly, the 25 µl reaction mixture consisted of 12.5 µl 2 × PyroMark PCR master mix (Qiagen, Germany), 1 µl of LCGreen dye 10 × (final concentration 0.4 ×, Idaho Technology, USA), 0.1 µl of forward (bio-pBR5`.SE) and reverse primers (pBR-V1.AS; 0.4 µmol/l final concentration each) and 2 µl of DNA template. PCR was performed in a LightCycler 96 instrument under the following cycling parameters: initial denaturation at 95°C for 15 min; 45 cycles of 94°C for 30 s, 56°C for 30 s and 72°C for 30 s; followed by final extension at 72°C for 10 min. Pyrosequencing was performed on a PyroMark Q96 ID instrument (Qiagen, Germany) using the PyroMark Gold Q96 Reagents (Qiagen, Germany) following manufacturer’s instructions. Briefly, 20 µl of amplified DNA products were mixed with 3 µl Streptavidin sepharose beads (GE Healthcare Life Sciences, Sweden), 40 µl of binding buffer (Qiagen, Germany) and 17 µl of deionised water, followed by shaking at 1,400 rpm for at least 10 min. Then, the immobilized complex of PCR products-Streptavidin sepharose beads was captured using a Vacuum Prep Workstation (Qiagen, Germany). Single strand purification was achieved by successive washing with 70% ethanol, denaturation buffer (Qiagen, Germany) and washing buffer (Qiagen, Germany), for 10 s each. Through this step, the non-biotinylated strand was dissociated and discarded. The sepharose beads with biotinylated strand were then released into a 96-well plate that was pre-filled with 40 µl of sequencing primer diluted in annealing buffer at 0.4 mmol/l final concentration. The plate was incubated at 80°C for 2 min, followed by slow cooling to room temperature. Then the plate was loaded into a PyroMark Q96 ID system instrument with the PyroMark Q96 application software 1.0 (Qiagen, Germany) set on 25 cycles of ATCG dispenses. The resulting sequences were compared with sequences in the Genbank database using BLAST tool provided on-line by NIH (http://blast.ncbi.nlm.nih.gov/Blast.cgi), Basic Local Alignment Search Tool.

Performance of the two identification techniques was evaluated using three parameters. Identification depth was calculated as the percentage of isolates identified to species level by a particular technique (isolates identified to species level/total number of isolates). Sensitivity was calculated as the percentage of unambiguous results of identification (either species or genus), *i.e.* number of unambiguous identification results (species or genus level) divided by the total number of isolates tested by particular technique. Specificity was calculated as the percentage of isolates where incorrect identification was excluded (no incorrect species or genus identification was issued by a particular technique). The correctness of the identification was indicated by the MALDI Biotyper score and e-value of pyrosequencing.

## RESULTS AND DISCUSSION

Results of identification level achieved by the tested techniques are summarized in **Table 1**. We were not able to identify 8 clinica l isolates by either of the techniques. The specificity of both methods reached 100%, *i.e.* if any of them issued a result, it was never incorrect. However, their performance in terms of identification depth was fairly different. Pyrosequencing was able to reach the species level in 43.2% of isolates only, whereas MALDI-TOF was clearly superior by achieving species level identification in 96.8% of the isolates tested. The overall sensitivity values also clearly underlined the superiority of MALDI-TOF MS with 96.8% compared to 85.1% achieved by pyrosequencing (some of the isolates were identified neither to species, nor genus level, just higher taxa, which was evaluated as insufficient and thus decreased the sensitivity value). Obviously, variability of the initial part of V1 region in the 16S rDNA detected by pyrosequencing is not sufficient enough for broad-range identification of several bacterial species. In our study, length of successfully sequenced portion of the V1 region varied extensively, namely between 9 and 144 nucleotides with median of 48 nucleotides. Then, insufficient depth and sensitivity of identification should be due to the shorter read length, as reported also by others [8, 9, 10]. According to some authors, combined pyrosequencing results from the regions V1 and V3 or V6 could be more beneficial [7, 10]. Unfortunately, this would further increase the running costs of pyrosequencing compared to MALDI-TOF MS in routine use.

On the other hand, due to an extensive database of DNA sequences available in GenBank, pyrosequencing proved to be more suitable for the identification of rare species not yet included in the commercial MALDI-TOF MS database. For example, MALDI-TOF MS completely failed to identify *Asaia bogorensis*, which was successfully and unequivocally achieved by pyrosequencing. Notably, this species was recovered on *Burkholderia cepacia* selective agar, indicating possible initial infection by a serious pathogen in cystic fibrosis settings. When MALDI-TOF identification fails in such case, correct identification should be achieved without unnecessary delay. Then, pyrosequencing represents a rapid and economic alternative to full Sanger sequencing. Interestingly, *Asaia* sp. was recently described to contaminate and decay fruit-flavoured drinks [11]. Most probably, this species just transiently colonized patients’ respiratory pathways after drinking spoiled beverages, because later, it was never recovered again. Thus, pyrosequencing resolved this puzzling culture result without the need to start a *Burkholderia* sp. eradication attempt. Further, *Variovorax* sp. that was already detected in hospital environment in CF patient samples [12] also posed an insurmountable problem for MALDI-TOF, whereas V1 pyrosequencing was able to identify the genus. MALDI-TOF also performed inferior to pyrosequencing in *Granullicatella adiacens*, where 6 of the 13 strains were not identified by MALDI-TOF MS to species level, whereas all of them were successfully identified by V1 pyrosequencing. Similarly, one *Staphylococcus pasteuri* strain, and, more importantly, one *Pseudomonas aeruginosa* strain were not identified to species level by MALDI-TOF MS in contrast to V1 pyrosequencing.

**Table 1:** List of species and results of identification by MALDI-TOF MS and pyrosequencing.

Species	Number of isolates	Highest level of identification achieved	
		MALDI-TOF MS	pyrosequencing
*Acinetobacter baumannii, calcoaceticus, pittii, ursingii*	6	species	genus
*Actinomyces oris*	5	species	species
*Aggregatibacter aphrophilus, segnis*	12	species	group 1*
*Achromobacter ruhlandii, spanius, xylosoxidans*	12	species	genus
*Asaia bogorensis*	1	none	species
*Bacillus licheniformis*	2	species	none
*Bacillus pumilus*	3	species	species
*Bordetella bronchiseptica*	3	species	genus
*Brevibacterium casei*	2	species	none
*Burkholderia cepacia complex (B. cenocepacia, cepacia sensu stricto, multivorans)*	28	species	complex
*Citrobacter freundii*	2	species	species
*Corynebacterium amycolatum, aurimucosum, durum, matrochotii, propinquum, pseudodiphteriticum, striatum*	20	species	genus
*Cupriavidus metallidurans*	4	species	species
*Delftia acidovorans*	2	species	species
*Eikenella corrodens*	1	species	species
*Enterobacter asburiae, aerogenes, cloacae, kobei, ludwigii*	11	species	group 2**
*Enterococcus faecalis*	5	species	species
*Erwinia persicina*	1	species	genus
*Escherichia coli*	4	species	species
*Gemella haemolysans, sanguinis*	16	species	none
*Granulicatella adiacens*	13	species (7)/none (6)	species
*Haemophilus haemolyticus, influenzae*	83	species	group 1*
*Haemophilus parahaemolyticus*	1	species	none
*Haemophilus parainfluenzae*	127	species	species
*Haemophilus pittmaniae*	1	species	genus
*Chryseobacterium hominis*	1	species	none
*Kingella denitrificans*	5	species	species
*Lactobacillus casei, paracasei*	8	species	genus
*Lactobacillus fermentum, plantarum, rhamnosus, salivarius*	16	species	species
*Lactobacillus gasseri*	1	species	genus
*Lactobacillus kimchii*	1	species	none
*Lactococcus lactis*	1	species	none
*Micrococcus luteus*	1	species	species
*Moraxella catarrhalis, liquefaciens, nonliquefaciens*	15	species	genus
*Neisseria flavescens, macacae*	4	species	genus
*Neisseria oralis*	1	none	species
*Ochrobactrum tritici*	2	species	none
*Pannonibacter phragmitetus*	2	species	species
*Proteus mirabilis, vulgaris*	7	species	group 1*
*Pseudomonas aeruginosa*	127	species (126)/none (1)	species
*Pseudomonas grimontii, mendocina, veronii*	16	species	genus
*Ralstonia pickettii*	3	species	species
*Rhizobium radiobacter*	1	species	family
*Rhodococcus kroppenstedtii*	1	species	genus
*Rothia aeria*	8	species	genus
*Rothia dentocariosa, mucilaginosa*	26	species	species
*Serratia marcescens*	3	species	species
*Staphylococcus aureus, cohnii, epidermidis, hominis*	70	species	species
*Staphylococcus pasteuri*	1	none	species
*Stenotrophomonas maltophilia*	19	species	species
*Streptococcus agalactiae, infantis, massiliensis, oligofermentans, parasanguinis*	51	species (48)/none (3)	species
*Streptococcus anginosus, constellatus, dysgalactiae, gordonii, intermedius, mitis, oralis, peroris, pneumoniae, pyogenes, salivarius, sanguinis, vestibularis*	351	species (339)/none (12)	genus (350)/none (1)
*Streptococcus mutans*	5	species	none
*Streptomyces scabiei, griseus*	2	species	genus
*Variovorax*	2	none	genus
Not identified	8	none	none

^*^Group 1 included genera *Aggregatibacter*, *Haemophillus* (*haemolyticus*and*influenzae*) and *Proteus*. ^**^Group 2 included genera *Enterobacter*and *Klebsiella*.

## CONCLUSIONS

Generally, MALDI-TOF MS turned out to be the best suitable technique in routine bacterial identification for its easy and economic performance and low labour costs. In contrast, V1 region pyrosequencing failed as general routine technique, although it offered an economic opportunity for rare species identification not yet included in the database of protein profiles. With time, on the other hand, extended length reading at lower costs can be expected due to technical improvements in future, which may improve the performance and application potential of pyrosequencing. For the moment, however, pyrosequencing could be recommended as the method of choice only in situations where MALDI-TOF MS goes wrong.
